# The association between electronic cigarettes, sleep duration, and the adverse cardiovascular outcomes: Findings from behavioral risk factor surveillance system, 2020

**DOI:** 10.3389/fcvm.2022.909383

**Published:** 2022-10-06

**Authors:** Xingyou Liu, Zhichao Yuan, Yuelong Ji

**Affiliations:** ^1^First School of Clinical Medicine, Yunnan University of Chinese Medicine, Kunming, China; ^2^Department of Maternal and Child Health, School of Public Health, Peking University, Beijing, China

**Keywords:** sleep duration, electronic cigarettes, cardiovascular diseases, young adults, joint effect

## Abstract

The joint effect of electronic cigarette smoking and insufficient sleep duration on cardiovascular disease (CVD) was unclear. This cross-sectional study aimed to evaluate the association between electronic cigarettes, sleep duration, and risk of CVD among American adults. The participants who completed the survey from the behavioral risk factor surveillance system in 2020 were included in this study. The status of electronic cigarette smoking was divided into never, former, and current use. The duration of sleep was categorized into insufficient (<6 h), appropriate (6–9 h), and excessive (>9 h) groups. The CVD group was defined as a patient having any of the following conditions: heart attack, coronary heart disease, or stroke according to self-report. The multivariate logistic regression model was adopted to determine the association between electronic cigarettes, sleep duration, and the risk of CVD. Sensitivity analyses were performed to assess the joint effects on the risk of CVD subtypes, including heart attack, coronary heart disease, and strokes, respectively. Subgroup analyses were performed to estimate the joint effects within the stratum of the age group. The total number of participants included in the present study was 253,561. Of which, 22,908 patients had CVD. In total, 61,293 participants had previously or currently used electronic cigarettes and 37,429 participants had inappropriate sleep duration. Former electronic cigarette users had a 10.8% increased risk of having CVD (OR = 1.108, 95% CI: 1.001–1.227) compared to users who never had electronic cigarettes. Insufficient and excessive sleep durations are associated with increased risks of CVD (OR = 1.592, 95% CI: 1.460–1.735; OR = 1.523, 95% CI: 1.320–1.758). The participants with current vaping status and lack of sleep had a 159.6% increased risk of CVD (OR = 2.596, 95% CI: 1.810–3.723). Sensitivity analyses found similar joint effects of current vaping and insufficient sleep on the risk of heart attack, coronary heart attack, and stroke. The subgroup analyses across each age stratum found that the middle-aged group is most vulnerable to the joint effect of current vaping and insufficient sleep. This study found that both current vaping and inappropriate sleep duration were associated with CVD. Additionally, there was a significant joint effect of current vaping and insufficient sleep on the risk of CVD, especially for middle-aged participants.

## Introduction

Cardiovascular disease (CVD) is a group of lifestyle-related disorders of the heart and the blood vessels (1). The data from the American Heart Association (AHA) showed that over 26 million adults are suffering from CVD in the US (2, 3). CVD is the leading cause of death globally, which lays a huge health burden and leads to wealth loss to all aspects of society (2). It is important to note that CVD is still a preventable disease. Epidemiological studies showed that above 90% of the risk of CVD could be avoided by a healthy lifestyle and timely and appropriate medical care (4). Studies also illustrated that lifestyle factors, such as a fatty diet, tobacco use, obesity, and physical inactivity, are associated with the mortality of CVD (5–9).

Traditional combustible cigarette smoking is an eminent risk factor for the development of CVD (10). Due to its highly addictive vapor, it is extremely hard to quit cigarette smoking (11). Since 2004, electronic cigarettes have been invented to replace traditional combustible cigarettes (12). Electronic cigarettes are made up of three parts: a plastic tube, an electronic heater, and a cartridge containing a liquid solution of propylene glycol with or without nicotine (13). Due to the low cost and good portability, the vaping population of electronic cigarettes has grown drastically in the US. A recent study indicated that roughly 8 million American adults have ever used electronic cigarettes (14). Another study showed that more than half of the users of electronic cigarette were young adults. Electronic cigarettes also gained huge popularity among the users of traditional combustible cigarettes, which is used as a smoking cessation aid. Unlike traditional nicotine replacement therapies, vaping mimics the action of combustible cigarette smoking while avoiding the release of harmful tar and carbon monoxide, which were typically released from the combustion of traditional nicotine cigarettes (15). However, the safety and efficacy of vaping electronic cigarettes still remain unsettled in the past decade.

Besides electronic cigarettes, sleep duration and quality are also the major risk factors for the dysfunction of the cardiovascular and cerebrovascular systems (16). Sleep is an important biological behavior process, accounting for a large part of life. Mounting studies indicated that multiple biological processes happen during sleep, such as metabolism (17), appetite regulation (18), immune reaction (19), hormone balance (20), and emotion control (21), which are essential for physical and mental wellbeing. In addition to CVD, inappropriate sleep condition is also associated with a group of metabolic or psychological outcomes such as obesity (22), diabetes (23), cancer (24), and depression (25). From 1985 to 2012, the number of American adults with <6 h of total sleep time per day increased from 38.6 to 70.1 million (22). Meanwhile, sleep disrupting factors such as increased screen time and unhealthy lifestyles are on the rise for the past decades. Therefore, in assessing the risk of CVD, it is impossible to ignore the persistent decline in sleep duration and quality. A recent study showed that smoking is significantly associated with insomnia and a short duration of sleep (26). However, there is no study that simultaneously focused on the associations between electronic cigarettes, sleep duration, and CVD.

This study estimated the relationship between electronic cigarettes, sleep time, and CVD based on the latest national survey data. Furthermore, we assessed the joint effect of vaping and duration of sleep on the risk of CVD and its subtypes, including heart attack, coronary heart disease, and stroke.

## Materials and methods

### Study design and participants

In this cross-sectional study, participants who took the behavioral risk factor surveillance system (BRFSS), which is the largest national health survey conducted by the Centers for Disease Control and Prevention (CDC), were selected. BRFSS collected information from the residents of each state regarding their health-related risk behaviors, chronic health conditions, and use of preventive services. The electronic cigarette module is an optional independent module that states opt to use based on their needs during the phone call survey. In the current study, we utilized the latest data on electronic cigarettes, sleep duration, and CVD, gathered from the survey conducted in 2020 (*N* = 274,767). We further excluded the respondents with missing data on potential confounder variables, including age, race, BMI, chewing tobacco use, medical history of diabetes, depression, and chronic obstructive pulmonary disease (COPD) (*N* = 21,206). A total of 253,561 participants were included in the final sample.

### Data collection

The history of electronic cigarette use was identified by the following question: *Have you ever used an e-cigarette or other electronic “vaping” product, even just one time, in your entire life?* Those who responded *no* were categorized as never electronic cigarette users. The respondents who answered *yes* were then asked, *Do you now use e-cigarettes or other electronic “vaping” products every day, some days, or not at all?* Respondents who answered “*not at all”* were considered former electronic cigarette users and those who answered *every day* or *some days* were categorized as current electronic cigarette users. In terms of the history of combustible cigarette use, the respondents were evaluated by the following question: *Have you smoked at least 100 cigarettes in your entire life?* The participants who answered *no* were defined as never combustible cigarette users. Those who responded *yes* were further asked: *Do you now smoke cigarettes every day, some days, or not at all?* The participants who reported *every day or some days* were defined as current combustible cigarette users. The time of sleep duration was collected from the question: *On average, how many hours of sleep do you get in 24 h?* The sleep duration of participants was classified into three categories according to the recommendation from the American Academy of Sleep Medicine (AASM) and the Sleep Research Society (SRS) (27). Insufficient sleep duration was defined as the sleep duration of <6 h in 24 h; appropriate sleep duration was defined as the sleep duration between 6 and 9 h in 24 h; excessive sleep duration was considered as the sleep duration above 9 h in 24 h (27). The participants having either insufficient or excessive sleep duration were further defined as having inappropriate sleep duration.

Sociodemographic variables, lifestyle, and disease history were collected from a standardized questionnaire, including sex (men and women), age (<35, 35–55, and ≥55 years), race (white only, black only, and other), physical activity (yes, no), chewing tobacco use (yes, no), education level (low, middle, and high), BMI (continuous), combustible smoking (never, former, and current), COPD (yes, no), depression (yes, no), and diabetes (yes, no). A low education level was considered as a grade <9. The middle education level was a grade between 9 and 12. A high education level was defined as a grade above 12. The chewing tobacco use was assessed according to the question: *Do you currently use chewing tobacco every day, some days, or not at all?* Respondents who answered *not at all* were determined as “No,” and the others were considered as “Yes.” The physical activity was determined by the question: *During the past month, other than your regular job, did you participate in any physical activities or exercises such as running, calisthenics, golf, gardening, or walking for exercise?* The history of COPD, depression, and diabetes was evaluated based on the question: *Were you ever told you had COPD, depressive disorder, or diabetes?* The questionnaire and detailed codebook could be obtained from the website link: https://www.cdc.gov/brfss/annual_data/2020/pdf/codebook20_llcp-v2-508.pdf. The adverse cardiovascular outcomes (CVD) of heart attack, coronary heart disease, and stroke were determined by asking the following questions: *Has a doctor, nurse, or other health professional ever told you that you had a heart attack/coronary heart disease/ stroke?*

### Ethics

The study was based on the American national telephone survey (BRFSS, https://www.cdc.gov/brfss/index.html).

### Statistical analysis

Considering that the data on electronic cigarettes were collected by different versions of the questionnaire, we pooled the data by the appropriate weighting methodology as published by the CDC. For instance, New York state used two versions of questionnaires to collect information on electronic smoking; therefore, two datasets need to be pooled. First, we used the sample size from each dataset divided by the summed sample size of these two datasets to get the corresponding proportion for each one. Then, we got the pooled data by recalculating the survey weight by multiplying the old survey weight with their dataset's corresponding proportion. The detailed pooling process could be obtained from https://www.cdc.gov/brfss/annual_data/2020/pdf/Complex-Smple-Weights-Prep-Module-Data-Analysis-2020-508.pdf. The weighting method ensured that the pooled data retained national representativeness and were comparable among different states.

In the univariate comparison, the characteristics of the categorized participants were analyzed by the chi-squared test with Rao and Scott's second-order correction. The characteristics of the continuous participant were analyzed by the Wilcoxon rank-sum test. The multivariate logistic regression model was adopted to assess the association between electronic cigarettes, combustible cigarettes, sleep duration, and CVD, adjusted by sex, age, race, education levels, physical activity, chewing tobacco use, combustible cigarette smoking, BMI, diabetes, depression, and COPD. Furthermore, we estimated the association between sleep duration and the risk of CVD in participants who were currently both electronic and combustible smokers by the multivariate logistic regression model adjusted for the same covariables. This study performed 2 sensitivity analyses to evaluate the robustness of the associations. The first sensitivity analysis was conducted after excluding the participants who were current and former combustible cigarette smokers, which controlled the bias from the effect of combustible smoking. The second sensitivity analysis evaluated the joint effect of electronic cigarettes and sleep duration on CVD subtypes, including heart attack, coronary heart disease, and stroke. Additionally, we conducted a subgroup analysis to examine the joint effect of vaping and sleep conditions on the risk of CVD across different ages, sex, and race strata. The age strata were categorized into young (<35 years old), middle-aged (35–55 years old), and old age (older than 55 years).

All multivariate logistic regression results are presented as odds ratios (OR) with a 95% confidence interval (CI). A 2-sided *p* < 0.05 was defined as a significant difference. All analyses were conducted using the survey package in R software (version 4.0.0).

## Results

### Characteristics of the participants

**Figure 1:**
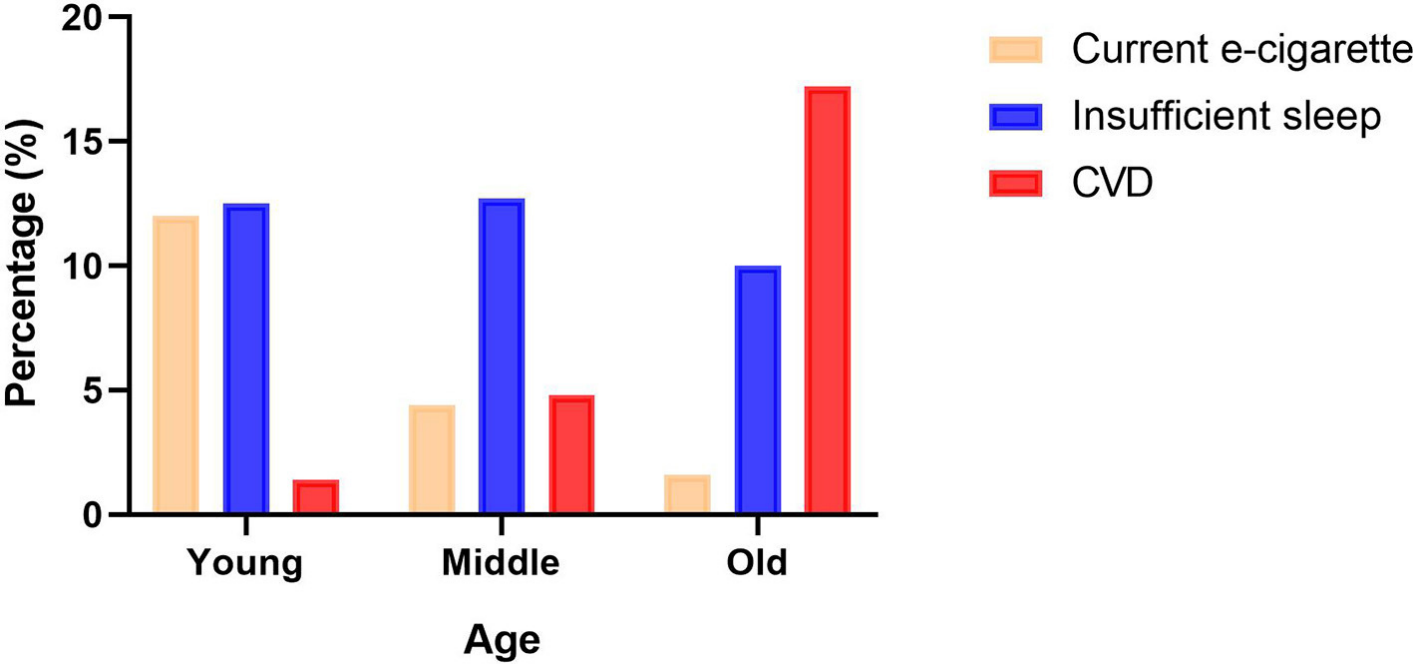
The prevalence of current electronic cigarettes, insufficient sleep duration, and cardiovascular disease (CVD) in young, middle, and old adults. The orange column represents the prevalence of current electronic cigarettes; the blue column shows the prevalence of insufficient sleep duration; the red column indicates the prevalence of CVD.

This cross-sectional study included 253,561 American adults. Of which, 22,908 (9.0%) patients had CVD. Nearly, one-third of participants were below 35 years, and over two-thirds of participants were White. Table 1 presents the sociodemographic features and disease history. A total of 61,293 (24.2%) participants were former or current users of electronic cigarettes and a total of 37,429 (14.7%) participants had inappropriate sleep duration. The percentage of diabetes in patients with CVD was approximately two times higher than the percentage in non-CVD participants (31.3 vs. 10.7%, *P* < 0.001). The percentages of COPD and depression were also higher in participants with CVD compared with non-CVD participants (22.0 vs. 5.4%, *P* < 0.001; 26.7 vs. 18.8%, *P* < 0.001). We also observed a lower percentage of physical activity and a higher BMI in patients with CVD (both *P* < 0.001). In addition, there is an increasing trend of CVD prevalence with aging. In contrast, the prevalence of current electronic cigarette smoking status decreased with aging (4.2, 16.7, and 79.1%). The insufficient sleep duration was stable across different age groups (Figure 1).

**Table 1:** Sociodemographical characteristics, electronic cigarette use, and sleep duration of the study population.

**Characteristic**	**Overall** ***N* (%)**	**Non-CVD subjects** ***N* (%)**	**CVD subjects** ***N* (%)**	***P*-Value**
	253,561	230,653	22908	
**Sex**				
Women	128,642 (50.7%)	118,827 (51.5%)	9,815 (42.8%)	
Men	124,919 (49.3%)	111,826 (48.5%)	13,093 (57.2%)	<0.001
**Age**				
<35 years	69,133 (27.3%)	68,182 (29.6%)	951 (4.2%)	
35–55 years	79,457 (31.3%)	75,626 (32.8%)	3,831 (16.7%)	
≥55 years	104,971 (41.4%)	86,845 (38.6%)	18,126 (79.1%)	<0.001
**Race**				
White only	167,763 (66.2%)	150,619 (65.3%)	17,144 (74.8%)	
Black only	31,884 (12.6%)	29,187 (12.7%)	2,697 (11.8%)	
Other	53,914 (21.2%)	50,847 (22.0%)	3,067 (13.4%)	<0.001
**Education levels**				
Low	7,842 (31.0%)	6,774 (3.0%)	1,068 (4.7%)	
Middle	92,966 (36.7%)	82,891 (35.9%)	10,075 (44.0%)	
High	152,753 (60.3%)	140,988 (61.1%)	117,65 (51.3%)	<0.001
**Physical activity**				
Yes	193,948 (76.5%)	179,842 (78.0%)	14,106 (61.6%)	
No	59,613 (23.5%)	50,811 (22.0%)	8,802 (38.4%)	<0.001
**Chewing tobacco use**				
Yes	9,157 (3.6%)	8,324 (3.6%)	833 (3.6%)	
No	2,444,404 (96.4%)	222,329 (96.4%)	22,075 (96.4%)	0.911
**Combustible cigarettes smoking**				
Never	151450 (59.7%)	141,954 (61.5%)	9,496 (41.5%)	
Former	62,823 (24.8)	53,901 (23.4%)	8,922 (38.9%)	<0.001
Current	39,288 (15.5%)	34,798 (15.1%)	4,490 (19.6%)	
**Diabetes**				
Yes	31,817 (13.5%)	24,637 (10.7%)	7,180 (31.3%)	
No	221,744 (86.5%)	206,016 (89.3%)	15,728 (68.7%)	<0.001
**COPD**				
Yes	17,425 (6.9%)	12,375 (5.4%)	5,050 (22.0%)	
No	236,136 (93.1%)	218,278 (94.6%)	17,858 (78.0%)	<0.001
**Depression**				
Yes	49,444 (19.5%)	43,334 (18.8%)	6,110 (26.7%)	
No	204,117 (80.5%)	187,319 (81.2%)	16,798 (73.3%)	<0.001
BMI, mean (SD)	28.0 (7.0)	28.0 (7.0)	30.0 (7.0)	<0.001
**Electronic cigarettes smoking**				
Never	192,268 (75.8%)	173,476 (75.2%)	18,801 (82.1%)	
Former	47,843 (18.9%)	44,432 (19.3%)	3,411 (14.9%)	<0.001
Current	13,450 (5.3%)	12755 (5.5%)	696 (3.0%)	
**Sleep duration**				
Missing (NA)	11,667 (4.6%)	NA	NA	
Insufficient (<6 h per day)	27,929 (11.0%)	24,276 (10.5%)	3,653 (15.9%)	
Appropriate (6–9 h per day)	204,465 (80.6%)	188,192 (81.6%)	16,273 (71.0%)	<0.001
Excessive (>9 h per day)	9,500 (3.7%)	7,815 (3.3%)	1,685 (7.4%)	

CVD, cardiovascular diseases including heart attack, coronary heart disease, and stroke; COPD, chronic obstructive pulmonary disease; BMI, body mass index; N (%), number (percentage); NA, not available. Sociodemographic variables, lifestyle, and disease history were collected from a standardized questionnaire including sex, age, race, physical activity, education level, BMI, combustible cigarette smoking, COPD, depression, and diabetes. The questionnaire could be obtained from the website link (https://www.cdc.gov/brfss/annual_data/2020/pdf/codebook20_llcp-v2-508.pdf).

The categorized variables were analyzed by the chi-squared test with Rao and Scott's second-order correction. The continuous variables were analyzed by the Wilcoxon rank-sum test.

### Association between smoking, sleep duration, and CVD

Table 2 shows a 10.8% increased odds of having CVD (OR = 1.108, 95% CI: 1.001–1.227) for former electronic cigarettes users and a 17.0% increased odds (OR = 1.170, 95% CI: 0.969–1.412) for current electronic cigarettes users compared to users who never had electronic cigarettes. Similarly, former and current combustible cigarette smokers both significantly increased the odds of CVD (OR = 1.346, 95% CI: 1.256–1.400; OR = 1.452, 95% CI: 1.332–1.582). In terms of sleep duration, we observed significantly increased odds of the risk of CVD for both insufficient sleep duration and excessive sleep duration groups compared to the participants with appropriate sleep (OR = 1.592, 95% CI: 1.460–1.735; OR = 1.523, 95% CI: 1.320–1.758, respectively). A significant association was observed between insufficient sleep duration and the risk of CVD in participants who were currently both electronic and combustible smokers (OR = 2.330, 95% CI: 1.397–3.887, Supplementary Table 1). However, a non-significant association was found between excessive duration and the risk of CVD (OR = 1.317, 95% CI: 0.462–3.761, Supplementary Table 1). In terms of CVD subtypes, we found that inappropriate sleep duration was positively associated with the risk of heart attack, coronary heart disease, and stroke. Electronic cigarettes were positively associated with the risk of heart attack and coronary heart disease (Supplementary Table 2).

**Table 2:** Association between smoking, sleep duration, and CVD.

**Characteristic**	***N* (%)**	**OR**	**95% CI**	***P*-Value**
**Electronic cigarettes use status**				
Never users	18,801 (9.8%)	Ref		
Former users	3,411 (7.1%)	1.108	(1.001–1.227)	0.047
Current users	696 (5.2%)	1.170	(0.969–1.412)	0.102
**Combustible cigarettes use status**				
Never users	9,496 (6.3%)	Ref		
Former users	8,923 (14.2%)	1.346	(1.256–1.400)	<0.001
Current users	4,490 (11.4%)	1.452	(1.332–1.582)	<0.001
**Sleep duration status**				
Appropriate (6–9 h per day)	16,273 (8.0%)	Ref		
Insufficient (6 <per day)	3,653 (13.1%)	1.592	(1.460–1.735)	<0.001
Excessive (>9 h per day)	1,685 (17.7%)	1.523	(1.320–1.758)	<0.001

CVD, cardiovascular disease; OR, odds ratio; CI, confidence interval; N (%), the number of CVD cases (percentage among corresponding strata). CVD as a composite variable was defined as heart attack, coronary heart disease, or stroke.

The ORs were adjusted by sex, age, race, education levels, physical activity, chewing tobacco use, combustible smoking, BMI, diabetes, depression, and COPD.

### The joint effect of electronic cigarettes with combustible cigarettes or sleep duration on CVD

**Figure 2:**
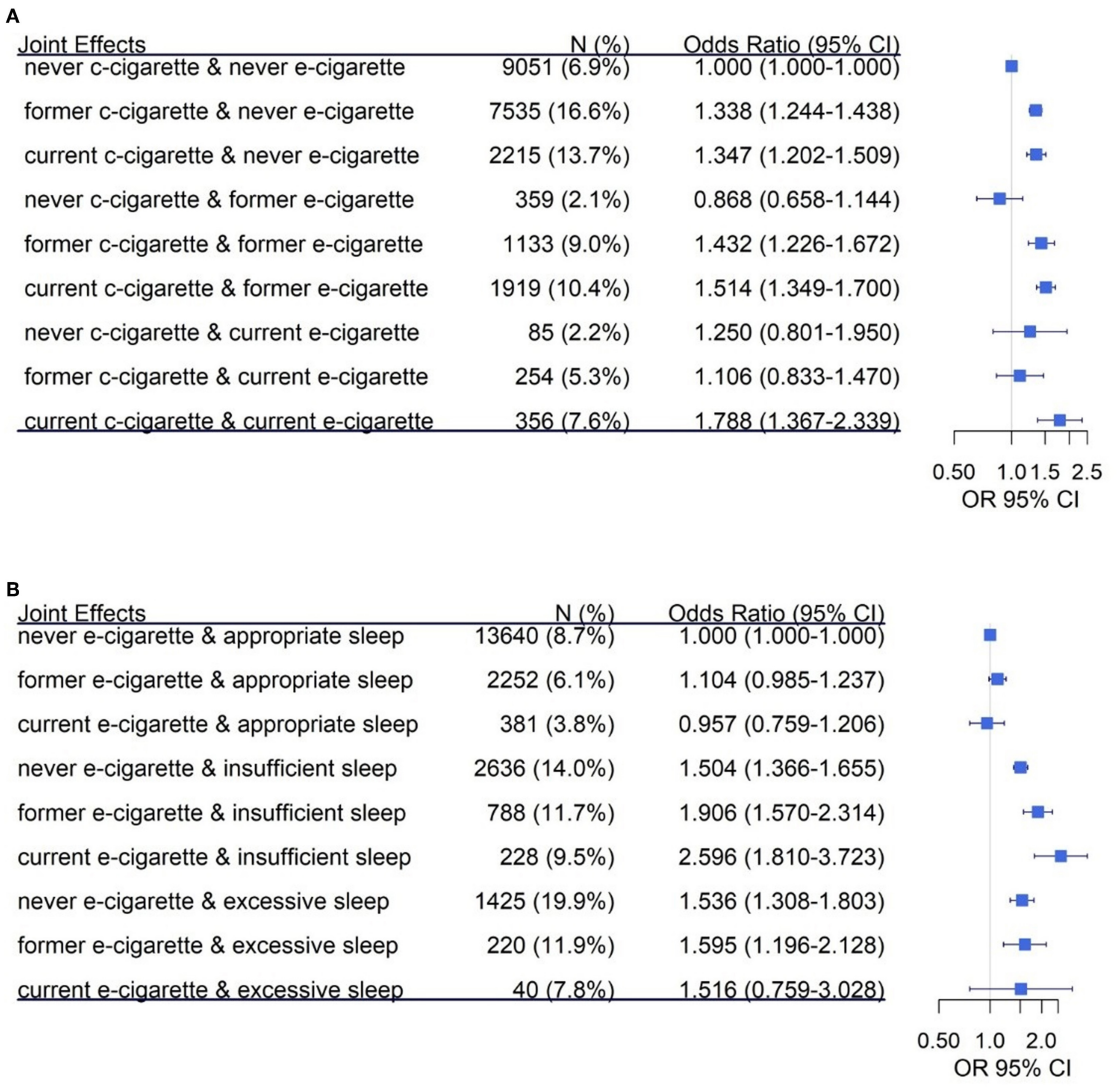
The joint effect of electronic cigarettes with combustible cigarettes or sleep duration on the risk of CVD. The joint effect was analyzed by multivariable logistical regression, which was adjusted by sex, age, race, education levels, physical activity, chewing tobacco use, combustible smoking, body mass index (BMI), diabetes, depression, and COPD. **(A)** The joint effect of electronic cigarettes and combustible cigarettes on CVD. The reference group **(A)** is defined as the participants who never smoked electronic and combustible cigarettes. **(B)** Presents the joint effect of electronic cigarettes and sleep duration on CVD. The reference group **(B)** is defined as the participants who never smoked electronic cigarettes and had appropriate sleep duration. c-cigarette, combustible cigarette; e-cigarette, electronic cigarette.

Figure 2A shows the joint effect of electronic cigarettes and combustible cigarettes on the risk of CVD. The participants who were both current electronic cigarette and combustible cigarette smokers had significantly elevated odds of CVD in comparison with the group who never smoked or vaped (OR = 1.788, 95% CI: 1.367–2.339). Additionally, the participants who were both former electronic cigarette and combustible cigarette smokers also increased the odds of CVD (OR = 1.432, 95% CI: 1.226–1.672). Participants with current vaping status and insufficient sleep duration had the highest odds of CVD (OR = 2.596, 95% CI: 1.810–3.723, Figure 2B) compared to participants who never vaped and had appropriate sleep duration. Meanwhile, we observed the significant joint effect of former electronic cigarette use and excessive sleep duration on the risk of CVD (OR = 1.595, 95% CI: 1.196–2.128). However, among participants with appropriate sleep duration, no significant associations were found for both former and current electronic cigarette use groups.

### Sensitivity analysis for the joint effect of electronic cigarettes and sleep duration

Supplementary Figure 1A shows a non-significant joint effect of current electronic cigarette smoking and insufficient sleep duration on the risk of CVD among the participants who never smoked combustible cigarettes, with a similar point estimate compared to the main analysis (OR = 2.111, 95% CI: 0.857–5.200). Supplementary Figures 1B–D indicates that the joint effect of current electronic cigarette smoking and insufficient sleep duration showed similar significantly positive associations with the three subtypes of cardiovascular outcomes (OR = 2.368, 95% CI: 1.839–3.049 for heart attack; OR = 3.059, 95% CI: 1.729–5.411 for coronary heart disease; OR = 2.807, 95% CI: 1.596–4.936 for stroke).

### Subgroup analysis for the joint effect of electronic cigarettes and sleep duration

Table 3 shows that the adults with current vaping status and insufficient sleep duration had significantly increased odds of CVD across the three age groups. The joint effect of electronic cigarettes and insufficient sleep was the highest among the middle-aged (OR = 3.149, 95% CI: 1.672–5.930). Across the different sex and race strata, the joint effect of current electronic cigarettes and insufficient sleep duration was higher among male participants (OR = 3.364, 95% CI: 1.981–5.710 for men vs. OR = 1.912, 95% CI: 1.220–2.995 for women) and was comparable between White and Black/other participants (OR = 2.612, 95% CI: 1.232–3.119 for White participants vs. OR = 2.612, 95% CI: 1.228–5.554 for Black/other adults, Supplementary Table 3). No significant interaction was found for these subgroup analyses.

**Table 3:** The joint effect of electronic cigarettes and sleep duration among different age groups.

**Joint effect on the risk of CVD**	***N* (%)**	**OR**	**95% CI**	***P*-Value**
**Young-Aged group (<35 years)**				
Never e-cigarettes and appropriate sleep duration	323 (1.0%)	Ref		
Former e-cigarettes and appropriate sleep duration	205 (1.2%)	0.915	(0.621–1.350)	0.655
Current e-cigarettes and appropriate sleep duration	66 (1.1%)	0.784	(0.460–1.337)	0.372
Never e-cigarettes and insufficient sleep duration	85 (2.2%)	1.484	(0.876–2.515)	0.142
Former e-cigarettes and insufficient sleep duration	79 (2.8%)	1.499	(0.899–2.501)	0.120
Current e-cigarettes and insufficient sleep duration	56 (3.7%)	1.988	(1.043–3.788)	0.037
Never e-cigarettes and excessive sleep duration	51 (3.8%)	3.357	(0.779–14.457)	0.104
Former e-cigarettes and excessive sleep duration	16 (1.8%)	1.347	(0.518–3.506)	0.541
Current e-cigarettes and excessive sleep duration	15 (4.4%)	2.998	(0.953–9.437)	0.061
**Middle-Aged group (35–55 years)**				
Never e-cigarettes and appropriate sleep duration	1756 (3.5%)	Ref		
Former e-cigarettes and appropriate sleep duration	697 (5.6%)	1.293	(1.022–1.636)	0.032
Current e-cigarettes and appropriate sleep duration	104 (4.0%)	0.899	(0.584–1.384)	0.631
Never e-cigarettes and insufficient sleep duration	518 (8.0%)	1.543	(1.244–1.915)	<0.001
Former e-cigarettes and insufficient sleep duration	357 (13.4%)	2.309	(1.688–3.160)	<0.001
Current e-cigarettes and insufficient sleep duration	108 (16.0%)	3.149	(1.672–5.930)	<0.001
Never e-cigarettes and excessive sleep duration	111 (8.5%)	1.536	(1.044–2.259)	0.029
Former e-cigarettes and excessive sleep duration	59 (12.0%)	1.893	(1.128–3.175)	0.015
Current e-cigarettes and excessive sleep duration	6 (6.3%)	0.705	(0.249–1.995)	0.510
**Old-Aged group (≥55 years)**				
Never e-cigarettes and appropriate sleep duration	11561 (15.3%)	Ref		
Former e-cigarettes and appropriate sleep duration	1350 (18.6%)	1.157	(1.013–1.322)	0.031
Current e-cigarettes and appropriate sleep duration	212 (16.4%)	1.153	(0.829–1.602)	0.398
Never e-cigarettes and insufficient sleep duration	2033 (23.9%)	1.502	(1.346–1.677)	<0.001
Former e-cigarettes and insufficient sleep duration	352 (28.5%)	1.640	(1.282–2.100)	<0.001
Current e-cigarettes and insufficient sleep duration	64 (27.8%)	1.960	(1.232–3.119)	0.004
Never e-cigarettes and excessive sleep duration	1263 (28.0%)	1.507	(1.293–1.756)	<0.001
Former e-cigarettes and excessive sleep duration	145 (30.4%)	1.559	(1.093–2.223)	0.014
Current e-cigarettes and excessive sleep duration	19 (24.5%)	1.242	(0.521–2.960)	0.624

CVD, cardiovascular disease; OR, odds ratio; CI, confidence interval, N (%), the number of CVD cases (percentage among corresponding strata). CVD as a composite variable was defined as heart attack, coronary heart disease, and stroke.

The ORs were adjusted by sex, race, education levels, physical activity, chewing tobacco use, combustible smoking, BMI, diabetes, depression, and COPD.

## Discussion

The present study found positive associations between electronic cigarette vaping, sleep time, and the risk of CVD. There was a consistent joint effect between current vaping and insufficient sleep on the risk of CVD and its subtypes, especially for middle-aged participants.

Over the past decades, electronic cigarette consumption has increased considerably in the US market (28). Considering the increasing prevalence and mortality of CVD, researchers started to focus on the potential linkage between the rising use of electronic cigarettes and the increased prevalence of cardiovascular disease. Several epidemiological studies showed positive associations between electronic cigarettes and CVD (29, 30) in line with our study. Additionally, our result showed that the participants who simultaneously smoked electronic and combustible cigarettes significantly increased the risk of CVD. The electronic cigarette was commonly marketed as a lower-risk alternative for combustible cigarettes, which attracted traditional smokers to attempt the novel experience or for smoking cessation. Our findings showed that both electronic and combustible cigarette smokers had an even higher risk of CVD compared to those who only smoke combustible cigarettes, which was also consistent with previous studies (31, 32). Thus, considering the strong adverse associations for both single and dual consumption, vaping electronic cigarettes can further increase the risk of CVD, irrespective of whether the smoker gradually or abruptly replaced smoking with vaping.

In terms of sleep duration, several studies observed the association between inappropriate sleep duration and adverse cardiovascular events. A recent mendelian randomization analysis indicated that there is a U-shaped effect of sleep duration on CVD based on data collected from 404,044 UK Biobank participants (33). Another US study showed that irregular sleep duration is associated with poorer microvascular health in young college students (34). It should be noted that inappropriate sleep patterns can also elevate the risk of CVD among young adults, which should have the lowest risk for CVD. In our study, the adverse effects of insufficient and excessive sleep duration were observed. Furthermore, we also found a significant joint effect between insufficient sleep duration and current electronic cigarette use among young adults. According to our sensitivity and subgroup analyses, we presented consistent positive associations between the combination of insufficient sleep duration and electronic cigarettes and the risk of CVD irrespective of age groups or subtypes of CVD. To the best of our knowledge, this is the first study to simultaneously focus on the effect of electronic cigarettes and the duration of sleep in a large sample with a nationwide representation.

Numerous studies explored the potential mechanisms underneath the individual effects of electronic cigarettes and sleep duration on the cardiovascular system. These findings help us to further explain the potential pathways of the joint effect of electronic cigarettes and sleep duration. Several pathophysiological mechanisms illustrated the effect of electronic cigarettes. First, high sympathetic nerve activation was observed in the electronic cigarette smokers. The study showed that electronic cigarettes increased the sympathetic tone and decreased the vagal tone, which was a specific pattern with high cardiovascular risk (35). Other studies found a sympathetic predominance in cardiac rhythm in both short-term and long-term users of electronic cigarettes, which induce elevated heart rate, blood pressure, and myocardial contractility (36). Second, electronic cigarettes activate platelets in circulation. Similar to traditional combustible cigarettes, one study indicated that exposure to electronic cigarettes enhanced platelet activation and subsequently increased thrombogenesis (37, 38). Additionally, the ultrafine particles in electronic cigarettes were also found to be associated with platelet activation (39). Third, a gradual increase in oxidative stress and inflammation was observed in acute electronic cigarette users, which is strongly associated with adverse cardiovascular events. This implies that inhalation of electronic cigarettes might cause vascular endothelial injury (40). There are some similar effects between sleep duration and electronic cigarettes on the pathophysiology of CVD. Insufficient sleep duration was also found to be associated with increased sympathetic activation, resulting in automatic dysfunction. In a sleep experimental study, sleep deprivation has been shown to disturb the automatic nervous system balance and increase heart rate and plasma norepinephrine, which is associated with the risk of CVD (41). Inflammation was regarded as another overlap mechanism of CVD by sleep duration and electronic cigarettes. For instance, a systematic review concluded that short sleep duration was significantly associated with elevated interleukin-6 (42) and C-reactive protein levels (43, 44).

There are some interesting findings that are worth to be further discussed. First, we observed a stronger association between insufficient sleep on the risk of CVD among current dual smokers compared to results based on the whole sample. This finding indicated that both combustible and electronic smokers are more vulnerable to poor sleeping routines. Second, we found that young adults who currently vaped and had insufficient sleep duration showed ~1-fold increased odds of CVD. The incidence of CVD increased with an increase in age (45). Young adults belong to the lowest risk group compared to older adults. However, young adults are the main population of electronic smokers. Furthermore, a previous study indicated that about 20% of young adults were suffering from insufficient sleep duration (46). Our finding indicated that people should not ignore the effect of vaping and irregular sleep on their cardiovascular health even when they are young. Third, according to our subgroup analysis among different age groups, we found that the joint effect was strongest among middle-aged adults. There are two aspects to illustrate this interesting finding. On the one hand, aging is usually accompanied by an increased cardiovascular burden, especially in the large arteries (47). Therefore, in comparison with young adults, middle-aged adults seem to have poorer tolerance to CVD with exposure to unhealthy lifestyles like electronic cigarettes and insufficient sleep duration. On the other hand, our study demonstrated that the prevalence of electronic cigarettes in middle-aged adults was significantly higher in comparison with the old adults. Thus, the relative higher exposure to electronic cigarettes might be another reason for this finding. Fourth, a stronger joint effect of current electronic smoking and insufficient sleep duration was observed in male adults compared with female adults. Previous study indicated that there were sex differences in electronic smoking (48). Therefore, the sex differences might explain this difference.

There are some strengths in the present study. First, we used the latest national data from the US with a large population to assess the associations between electronic cigarette smoking, inappropriate sleep duration, and the risk of CVD. Second, this study is the first to estimate the joint effect of electronic cigarettes and sleep duration on CVD. Third, it is the first time that the effect of vaping and irregular sleep on cardiovascular health is evaluated among young adults. The findings have health implications for young adults, who are the main population of electronic cigarette users. They need to pay attention to the potential harm from vaping and inappropriate sleep duration for their cardiovascular health. Some limitations also need to be noticed. First, our sleep duration and CVD information were obtained from the self-reported survey. The recalling bias might exist. More objective measurements for sleep and clinical diagnosis data should be used for future studies. Second, there are numerous different brands and types of electronic cigarettes on the market. In addition, we did not obtain the use history of electronic cigarettes, such as use dose and time. The effects of different types and lengths of vaping history still need to be studied in the future study. Third, other risk factors for CVD such as alcohol intake, noise, air pollution, and hypertension should be adjusted. However, due to the data source from BRFSS, we cannot adjust the effects of these risk factors in our present study. Fourth, the current study is cross-sectional. A cohort study is recommended to better explain the temporal relationship between vaping, sleep duration, and the risk of CVD.

## Conclusion

This study found that both vaping and inappropriate sleep duration were associated with CVD. Additionally, there was a significant joint effect of current vaping and insufficient sleep on the risk of CVD, especially for middle-aged participants.

## Data availability statement

Publicly available datasets were analyzed in this study. This data can be found at: https://www.cdc.gov/brfss/index.html.

## Ethics statement

Ethical review and approval was not required for this study in accordance with the local legislation and institutional requirements. Written informed consent was not required for this study in accordance with the local legislation and institutional requirements.

## Author contributions

YJ and ZY: conceptualization, methodology, and writing—review & editing. XL and ZY: data curation, formal analysis, investigation, and visualization. YJ: funding acquisition. XL: writing—original draft.

## Funding

This study was funded by startup funding from the Peking University Health Science Center (Grant No. BMU2021YJ045).

## Conflict of interest

The authors declare that the research was conducted in the absence of any commercial or financial relationships that could be construed as a potential conflict of interest.

## Publisher's note

All claims expressed in this article are solely those of the authors and do not necessarily represent those of their affiliated organizations, or those of the publisher, the editors and the reviewers. Any product that may be evaluated in this article, or claim that may be made by its manufacturer, is not guaranteed or endorsed by the publisher.

## Supplementary material

Supplementary Figure 1 The joint effect of electronic cigarettes and sleep duration for sensitivity analysis. **(A)** The joint effect after dropping the current and former combustible cigarette smokers to control the bias from the combustible cigarettes. **(B–D)** Indicates the joint effect of electronic cigarettes and sleep duration on the risk of heart attack, coronary heart disease, and stroke, respectively. All the joint effects were analyzed by multivariable logistical regression, which was adjusted by sex, age, race, education levels, physical activity, chewing tobacco use, combustible smoking, BMI, diabetes, depression, and COPD. The reference group was defined as the participants who never smoked electronic cigarettes and had appropriate sleep duration.

Supplementary Table 1 Association between sleep duration and CVD in current combustible and electronic cigarette smokers. CVD, cardiovascular disease; OR, odds ratio; CI, confidence interval; *N* (%), the number of CVD cases (percentage among corresponding strata). CVD as a composite variable was defined as heart attack, coronary heart disease, and stroke. The ORs were adjusted by sex, age, race, education levels, physical activity, chewing tobacco use, BMI, diabetes, depression, and COPD.

Supplementary Table 2 The effect of e-cigarettes and sleep duration on single outcomes. OR, odds ratio; CI, confidence interval; *N*(%), the number of CVD cases (percentage among corresponding strata). The ORs were adjusted by sex, age, race, education levels, physical activity, chewing tobacco use, combustible smoking, BMI, diabetes, depression, and COPD.

Supplementary Table 3 The joint effect of electronic cigarettes and sleep duration among different sex and race groups. CVD, cardiovascular disease; OR, odds ratio; CI, confidence interval; *N* (%), the number of CVD cases (percentage among corresponding strata). CVD as a composite variable was defined as heart attack, coronary heart disease, and stroke. The ORs were adjusted by sex, race, education levels, physical activity, chewing tobacco use, combustible smoking, BMI, diabetes, depression, and COPD.
