# Supplementary figures and images for: The association between electronic cigarettes, sleep duration, and the adverse cardiovascular outcomes: Findings from behavioral risk factor surveillance system, 2020
Source: Front Cardiovasc Med. 2022 Oct 6;9:909383. doi: 10.3389/fcvm.2022.909383 (PMC9582666; doi:10.3389/fcvm.2022.909383)

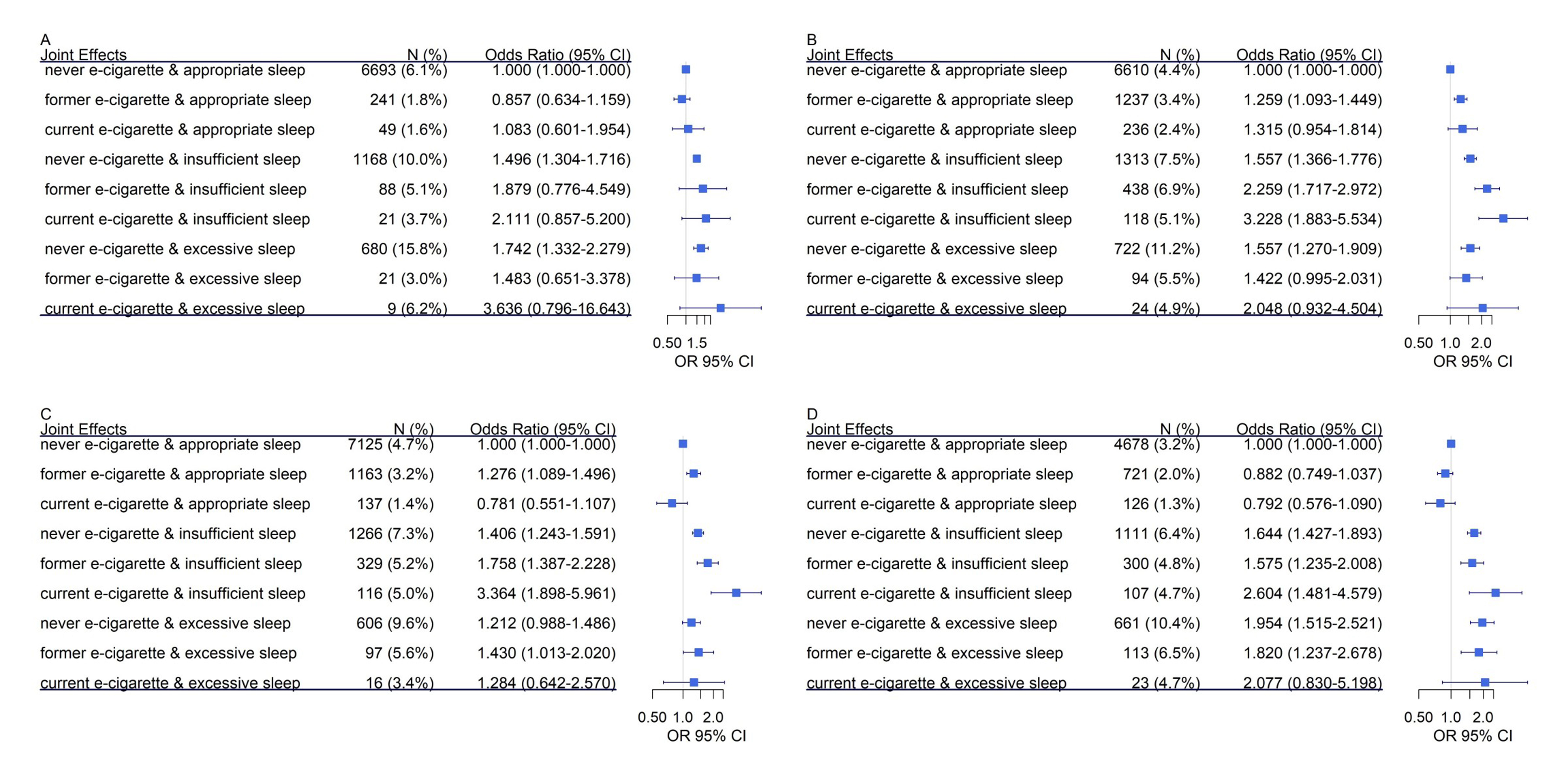

Supplement: Supplementary Figure 1 — The joint effect of electronic cigarettes and sleep duration for sensitivity analysis. (A) The joint effect after dropping the current and former combustible cigarette smokers to control the bias from the combustible cigarettes. (B–D) Indicates the joint effect of electronic cigarettes and sleep duration on the risk of heart attack, coronary heart disease, and stroke, respectively. All the joint effects were analyzed by multivariable logistical regression, which was adjusted by sex, age, race, education levels, physical activity, chewing tobacco use, combustible smoking, BMI, diabetes, depression, and COPD. The reference group was defined as the participants who never smoked electronic cigarettes and had appropriate sleep duration. [file Image_1.JPG]
