# Supplementary material for: The association between electronic cigarettes, sleep duration, and the adverse cardiovascular outcomes: Findings from behavioral risk factor surveillance system, 2020
Source: Front Cardiovasc Med. 2022 Oct 6;9:909383. doi: 10.3389/fcvm.2022.909383 (PMC9582666; doi:10.3389/fcvm.2022.909383)
Supplement: Supplementary Table 1 — Association between sleep duration and CVD in current combustible and electronic cigarette smokers. CVD, cardiovascular disease; OR, odds ratio; CI, confidence interval; N (%), the number of CVD cases (percentage among corresponding strata). CVD as a composite variable was defined as heart attack, coronary heart disease, and stroke. The ORs were adjusted by sex, age, race, education levels, physical activity, chewing tobacco use, BMI, diabetes, depression, and COPD. [file Data_Sheet_1.docx]

| Supplementary Table 1. Association sleep duration and CVD in current dual smokers | | | | |
| --- | --- | --- | --- | --- |
| Characteristic | N(%) | OR | 95% CI | P value |
| Sleep duration status |  |  |  |  |
| Appropriate (6-9h per day) | 188 (5.6%) | Ref |  |  |
| Insufficient (6< per day) | 138(13.3%) | 2.330 | (1.397-3.887) | 0.001 |
| Excessive (>9h per day) | 19(10.7%) | 1.317 | (0.462-3.761) | 0.605 |
| CVD: cardiovascular disease; OR: odds ratio; CI: confidence interval; N(%): the number of CVD cases (percentage among corresponding strata). CVD as composite variable was defined as heart attack, coronary heart disease and stroke.  The ORs were adjusted by sex, age, race, education levels, physical activity, chewing tobacco use, BMI, diabetes, depression, CODP. | | | | |

| Supplementary Table 2. The effect of e-cigarettes and sleep duration on single outcomes | | | | |
| --- | --- | --- | --- | --- |
| Characteristic | N(%) | OR | 95% CI | P value |
| **Heart attack** |  |  |  |  |
| Electronic cigarettes use status |  |  |  |  |
| Never users | 9170(5.0%) | Ref |  |  |
| Former users | 1828(4.0%) | 1.219 | (1.071-1.387) | 0.003 |
| Current users | 395(3.0%) | 1.493 | (1.151-1.938) | 0.003 |
| Sleep duration status |  |  |  |  |
| Appropriate (6-9h per day) | 8083(4.1%) | Ref |  |  |
| Insufficient (6< per day) | 1869(7.1%) | 1.645 | (1.460-1.854) | <0.001 |
| Excessive (>9h per day) | 840(9.7%) | 1.495 | (1.243-1.797) | <0.001 |
| **Coronary heart disease** |  |  |  |  |
| Electronic cigarettes use status |  |  |  |  |
| Never users | 9536(5.2%) | Ref |  |  |
| Former users | 1665(3.6%) | 1.228 | (1.070-1.408) | 0.003 |
| Current users | 296(2.3%) | 1.172 | (0.877-1.567) | 0.280 |
| Sleep duration status |  |  |  |  |
| Appropriate (6-9h per day) | 8426(4.3%) | Ref |  |  |
| Insufficient (6< per day) | 1710(6.6%) | 1.470 | (1.314-1.643) | <0.001 |
| Excessive (>9h per day) | 719(8.4%) | 1.207 | (1.008-1.445) | 0.040 |
| **Stroke** |  |  |  |  |
| Electronic cigarettes use status |  |  |  |  |
| Never users | 6914(3.8%) | Ref |  |  |
| Former users | 1203(2.6%) | 0.899 | (0.783-1.034) | 0.134 |
| Current users | 270(2.1%) | 1.040 | (0.781-1.384) | 0.788 |
| Sleep duration status |  |  |  |  |
| Appropriate (6-9h per day) | 5526(2.9%) | Ref |  |  |
| Insufficient (6< per day) | 1518(5.9%) | 1.726 | (1.526-1.953) | <0.001 |
| Excessive (>9h per day) | 798(9.3%) | 1.986 | (1.591-2.478) | <0.001 |
| OR: odds ratio; CI: confidence interval; N(%): the number of CVD cases (percentage among corresponding strata). The ORs were adjusted by sex, age, race, education levels, physical activity, chewing tobacco use, combustible smoking, BMI, diabetes, depression, CODP. | | | | |

| Supplementary Table 3. Joint effect of electronic cigarettes and sleep duration among different sex and race groups | | | | |
| --- | --- | --- | --- | --- |
| Joint effect on the risk of CVD | N(%) | OR | 95% CI | P value |
| Female |  |  |  |  |
| Never e-cigarettes and appropriate sleep duration | 5623(6.8%) | Ref |  |  |
| Former e-cigarettes and appropriate sleep duration | 912(5.7%) | 1.071 | (0.908-1.262) | 0.413 |
| Current e-cigarettes and appropriate sleep duration | 136(3.4%) | 0.814 | (0.569-1.165) | 0.261 |
| Never e-cigarettes and insufficient sleep duration | 1267(12.5%) | 1.531 | (1.337-1.753) | <0.001 |
| Former e-cigarettes and insufficient sleep duration | 397(12.7%) | 2.063 | (1.627-2.615) | <0.001 |
| Current e-cigarettes and insufficient sleep duration | 94(8.6%) | 1.912 | (1.220-2.995) | 0.004 |
| Never e-cigarettes and excessive sleep duration | 671(17.1%) | 1.642 | (1.337-2.017) | <0.001 |
| Former e-cigarettes and excessive sleep duration | 139(12.6%) | 1.917 | (1.329-2.766) | <0.001 |
| Current e-cigarettes and excessive sleep duration | 18(6.1%) | 1.645 | (0.591-4.577) | 0.340 |
| Male |  |  |  |  |
| Never e-cigarettes and appropriate sleep duration | 8017(10.7%) | Ref |  |  |
| Former e-cigarettes and appropriate sleep duration | 1340(6.3%) | 1.146 | (0.979-1.340) | 0.087 |
| Current e-cigarettes and appropriate sleep duration | 246(4.1%) | 1.073 | (0.792-1.453) | 0.650 |
| Never e-cigarettes and insufficient sleep duration | 1370(15.9%) | 1.483 | (1.293-1.701) | <0.001 |
| Former e-cigarettes and insufficient sleep duration | 391(10.8%) | 1.761 | (1.302-2.382) | <0.001 |
| Current e-cigarettes and insufficient sleep duration | 134(10.2%) | 3.364 | (1.981-5.710) | <0.001 |
| Never e-cigarettes and excessive sleep duration | 754(23.3%) | 1.442 | (1.131-1.839) | 0.003 |
| Former e-cigarettes and excessive sleep duration | 81(11.0%) | 1.157 | (0.780-1.716) | 0.467 |
| Current e-cigarettes and excessive sleep duration | 22(10.0%) | 1.502 | (0.615-3.672) | 0.372 |
| White adults |  |  |  |  |
| Never e-cigarettes and appropriate sleep duration | 10406(10.0%) | Ref |  |  |
| Former e-cigarettes and appropriate sleep duration | 1683(6.6%) | 1.052 | (1.013-1.322) | 0.378 |
| Current e-cigarettes and appropriate sleep duration | 296(4.0%) | 0.922 | (0.829-1.602) | 0.501 |
| Never e-cigarettes and insufficient sleep duration | 1785(16.9%) | 1.523 | (1.346-1.677) | <0.001 |
| Former e-cigarettes and insufficient sleep duration | 560(13.3%) | 1.944 | (1.282-2.100) | <0.001 |
| Current e-cigarettes and insufficient sleep duration | 157(10.0%) | 2.612 | (1.232-3.119) | <0.001 |
| Never e-cigarettes and excessive sleep duration | 1029(24.2%) | 1.578 | (1.293-1.756) | <0.001 |
| Former e-cigarettes and excessive sleep duration | 146(13.4%) | 1.420 | (1.093-2.223) | 0.032 |
| Current e-cigarettes and excessive sleep duration | 24(6.4%) | 1.342 | (0.521-2.960) | 0.401 |
| Black and other adults |  |  |  |  |
| Never e-cigarettes and appropriate sleep duration | 3233(6.1%) | Ref |  |  |
| Former e-cigarettes and appropriate sleep duration | 569(4.9%) | 1.288 | (0.965-1.718) | 0.086 |
| Current e-cigarettes and appropriate sleep duration | 86(3.4%) | 1.179 | (0.631-2.204) | 0.606 |
| Never e-cigarettes and insufficient sleep duration | 851(10.4%) | 1.441 | (1.191-1.742) | <0.001 |
| Former e-cigarettes and insufficient sleep duration | 228(9.1%) | 1.813 | (1.185-2.774) | 0.006 |
| Current e-cigarettes and insufficient sleep duration | 71(8.5%) | 2.612 | (1.228-5.554) | 0.013 |
| Never e-cigarettes and excessive sleep duration | 396(13.7%) | 1.537 | (1.072-2.205) | 0.020 |
| Former e-cigarettes and excessive sleep duration | 74(9.9%) | 2.140 | (1.227-3.735) | 0.008 |
| Current e-cigarettes and excessive sleep duration | 16(11.4%) | 1.242 | (0.521-2.960) | 0.309 |
| CVD: cardiovascular disease; OR: odds ratio; CI: confidence interval, N(%): the number of CVD cases (percentage among corresponding strata). CVD as composite variable was defined as heart attack, coronary heart disease and stroke.  The ORs were adjusted by sex, race, education levels, physical activity, chewing tobacco use, combustible smoking, BMI, diabetes, depression, CODP. | | | | |
